# Glasswing‐Butterfly‐Inspired Multifunctional Scleral Lens and Smartphone Raman Spectrometer for Point‐of‐Care Tear Biomarker Analysis

**DOI:** 10.1002/advs.202205113

**Published:** 2022-12-01

**Authors:** Vinayak Narasimhan, Radwanul Hasan Siddique, Un Jeong Kim, Suyeon Lee, Hyochul Kim, YoungGeun Roh, Yibing Michelle Wang, Hyuck Choo

**Affiliations:** ^1^ Department of Medical Engineering California Institute of Technology Pasadena CA 91125 USA; ^2^ Meta Vision Lab Samsung Semiconductor Inc. Pasadena CA 91101 USA; ^3^ Advanced Sensor Lab Samsung Advanced Institute of Technology Samsung Electronics Suwon Gyeonggi‐do 16678 South Korea

**Keywords:** bioinspired nanostructures, glasswing butterfly, multifunctional, scleral lens sensors, smartphone spectrometer, tear biomarkers

## Abstract

Augmenting contact lenses with sensing capabilities requires incorporating multiple functionalities within a diminutive device. Inspired by multifunctional biophotonic nanostructures of glasswing butterflies, a nanostructured scleral lens with enhanced optical, bactericidal, and sensing capabilities is reported. When used in conjunction with a smartphone‐integrated Raman spectrometer, the feasibility of point‐of‐care applications is demonstrated. The bioinspired nanostructures made on parylene films are mounted on the anterior and posterior side of a scleral lens to create a nanostructured lens. Compared to unstructured parylene, nanostructured parylene minimizes glare by 4.3‐fold at large viewing angles up to 80^o^. When mounted on a scleral lens, the nanostructures block 2.8‐fold more ultraviolet (UVA) light while offering 1.1‐fold improved transmission in the visible regime. Furthermore, the nanostructures exhibit potent bactericidal activity against *Escherichia coli*, killing 89% of tested bacteria within 4 h. The same nanostructures, when gold‐coated, are used to perform rapid label‐free multiplex detection of lysozyme and lactoferrin, the protein biomarkers of the chronic dry eye disease, in whole human tears using drop‐coating deposition Raman spectroscopy. The detection of both proteins in whole human tear samples from different subjects using the nanostructured lens produced excellent correlation with commercial enzyme‐based assays while simultaneously displaying a 1.5‐fold lower standard deviation.

## Graphical abstract

This work reports a multifunctional bioinspired nanostructured scleral lens. Inspired by biophotonic nanostructures on glasswing butterflies, bioinspired nanostructures made on parylene films are mounted on a scleral lens for enhanced optical, bactericidal, and sensing capabilities. The scleral lens sensor is used to detect lysozyme and lactoferrin in whole human tears for chronic dry eye disease management.

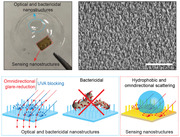

## 1 Introduction

The burgeoning contact lens (CL) market is predicted to reach US$19 billion by 2024 with 140 million users around the globe.^[^ ^1^, ^2^ ^]^ This has stimulated the development of several CL‐based sensors that provide more than traditional functions such as near‐/far‐sightedness and astigmatism correction.^[^ ^2^, ^3^, ^4^ ^]^ The most popular CL‐based sensors were first developed in both academia and industry for intraocular pressure (IOP)^[^ ^5^, ^6^, ^7^ ^]^ and glucose monitoring^[^ ^8^, ^9^, ^10^, ^11^ ^]^ to provide glaucoma and diabetes management, respectively. Following this development, the range of applications has grown to include dry eye disease,^[^ ^12^, ^13^ ^]^ xerophthalmia,^[^ ^7^ ^]^ color blindness,^[^ ^14^, ^15^ ^]^ and presbyopia management^[^ ^16^ ^]^ as well as a host of bio‐analyte/‐parameter monitoring such as lactate,^[^ ^17^ ^]^ pH,^[^ ^12^, ^18^ ^]^ temperature,^[^ ^19^ ^]^ electromagnetic wave shielding,^[^ ^8^ ^]^ and electroretinography.^[^ ^20^ ^]^ Table S1, Supporting Information, summarizes these technologies along with the various modes of operations used to achieve sensing. However, such CL‐based sensors along with their conventional counterparts are not designed to mitigate glare issues and ultraviolet (UV) transmission or to detect gram‐negative infections such as microbial keratitis.^[^ ^21^ ^]^ The use of nanoscale functionalization could provide a solution to each individual aforementioned problem, for example, UV/blue‐light blocking^[^ ^22^ ^]^ or antimicrobial activity^[^ ^23^, ^24^ ^]^ (Table S1, Supporting Information). However, with the ever‐increasing demand to incorporate more functionality within the same device footprint,^[^ ^25^, ^26^, ^27^ ^]^ a CL integrated with multiplexed biosensing, enhanced optical performance, and antimicrobial properties on a single platform could be of great value to personalized point‐of‐care medicine.

To integrate more functionality within the same device, researchers have sought inspiration from nature which showcases myriad examples of nanostructures with diverse functionalities for signaling, mating, camouflage, sterilization, thermoregulation, and other biological processes.^[^ ^28^, ^29^, ^30^, ^31^, ^32^, ^33^ ^]^ More specifically, these functionalities include enhanced omnidirectional optical effects,^[^ ^30^, ^32^ ^]^ bactericidal activity,^[^ ^34^, ^35^ ^]^ self‐cleaning,^[^ ^36^, ^37^ ^]^ and a variety of sensing capabilities.^[^ ^33^, ^38^ ^]^ Furthermore, such naturally occurring nanostructures have often served as an inspiration for engineered surfaces and sensors to perform similar functions.^[^ ^34^, ^39^, ^40^, ^41^, ^42^ ^]^ In the same vein, we previously demonstrated the integration of multifunctional nanostructures inspired by the *Chorinea faunus* butterfly onto a highly‐miniaturized intraocular pressure (IOP) sensor implant for Glaucoma monitoring.^[^ ^43^, ^44^, ^45^ ^]^ The integration of the bio‐inspired nanostructures greatly enhanced the in vivo optical performance and biocompatibility of the device.

**Figure 1:**
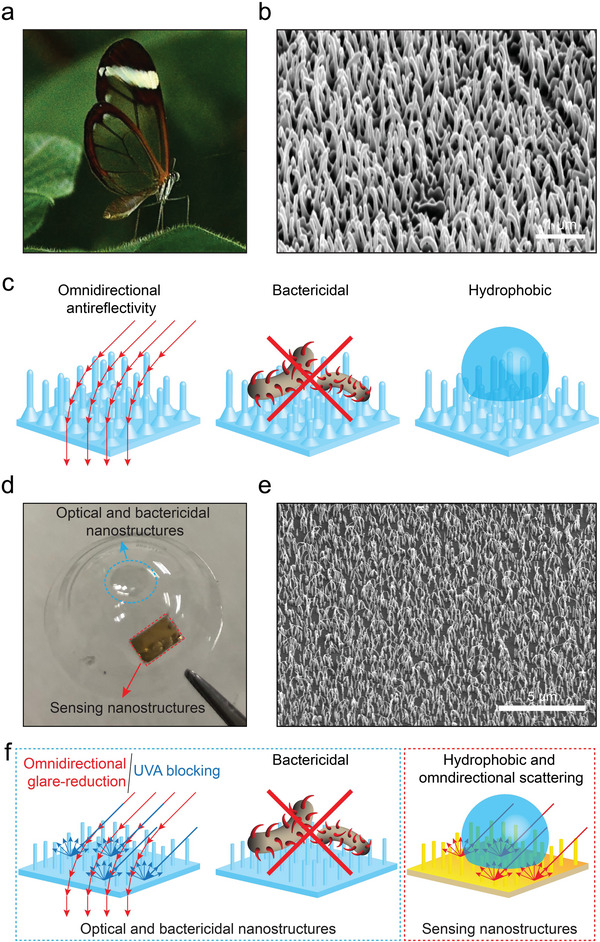
Glasswing butterfly‐inspired scleral lens sensor. a) The glasswing butterfly (*G. oto*). b) SEM image of high aspect‐ratio nanostructures on the *G. oto* wings. Scale bar: 1 µm. c) The multifunctionality of *G. oto* wings. d) Bioinspired nanostructured scleral lens. e) Both the optical and bactericidal region as well as the sensing region of the scleral lens consist of high aspect‐ratio parylene nanostructures. Scale bar: 5 µm. f) Multifunctionality of the nanostructures on the scleral lens.

Here, inspired by the multifunctional biophotonic nanostructures on the wings of glasswing butterflies (*Greta oto* and *C. faunus*), we report a scleral lens with multifunctional bioinspired nanostructures that provide optical, bactericidal, and sensing functionalities (**Figure** **1**). High aspect‐ratio random nanostructures on the wings of insects remarkably improve omnidirectional optical properties,^[^ ^32^ ^]^ bactericidal activity,^[^ ^34^, ^46^ ^]^ and self‐cleaning hydrophobicity (Figure 1a–c).^[^ ^36^, ^47^ ^]^ Using such multifunctional biophotonic nanostructures as bases, we have developed high aspect‐ratio nanopillars made of biocompatible parylene C and embedded them onto a scleral lens (Figure 1d–f). Like their natural counterparts, our bioinspired nanostructures display quasi‐random spatial distribution and significantly improve the optical properties of a scleral lens. Beyond optical properties, the bioinspired parylene nanostructures, much like other naturally‐occurring high aspect‐ratio nanostructures, display more potent bactericidal activity than unstructured parylene surfaces. Due to the co‐presence of the aforementioned optical and bactericidal properties, these nanostructures are incorporated in the optic zone on the anterior side of the scleral lens (Figure 1d). Outside of the optic zone and on the posterior side of the lens, we have designed and placed a small sensing region consisting of identical bioinspired parylene nanostructures conformally coated with gold (Au). The resulting hydrophobic Au‐coated bioinspired parylene nanostructures collect tear proteins through pinning of tear fluid and subsequent drying of the adherent tear drops. This process results in the concentration of tear proteins in the coffee ring, allowing highly sensitive rapid label‐free multiplexed detection of the two most dominant tear biomarkers for chronic dry eye disease – lysozyme and lactoferrin using drop‐coating deposition Raman spectroscopy (DCDRS).^[^ ^48^, ^49^ ^]^ The isotropic scattering and emission properties of the Au‐coated parylene nanostructures allow the use of a smartphone‐based detector to perform readouts from the scleral lens without an alignment issue which is a typical drawback of handheld optical readers.^[^ ^50^, ^51^ ^]^ As a result, the bioinspired nanostructures transform the conventional scleral lenses prescribed for dry eye disease patients into a versatile, multifunctional lens‐based sensor that could provide self‐monitoring of relevant biomarkers using smartphones. This would aid the development of highly effective personalized management of dry eye disease by quantitatively evaluating the efficacy of patient‐specific treatments.

## 2 Results and Discussion

### 2.1 Fabrication and Characterization of the Bioinspired Parylene Nanostructures

**Figure 2:**
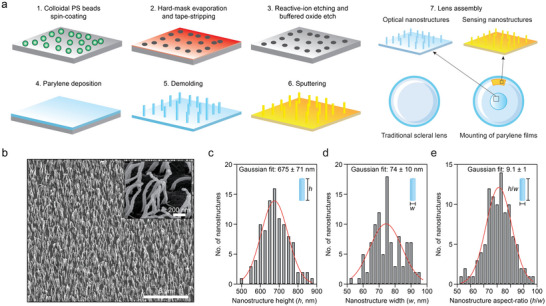
The bioinspired parylene nanostructures on scleral lens. a) Simple and scalable fabrication of the parylene nanostructures and integration on the scleral lens. b) High‐magnification SEM image of the nanostructures. Scale: 5 µm (inset scale: 200 nm). Similar to the nanostructures on glasswing butterflies, the c) height, d) width, and e) aspect‐ratio of the parylene nanostructures can be modeled using a Gaussian distribution.

Modeling after the biophotonic nanostructures on *G. oto* wings, we implemented high aspect‐ratio nanostructures with quasi‐random positioning and a random height distribution on parylene films.^[^ ^32^ ^]^ Parylene was chosen for its IC‐process compatibility, biocompatibility, mechanical stability as thin films, and optical clarity.^[^ ^52^, ^53^ ^]^ The fabrication process employs hole‐mask colloidal lithography and anisotropic etching on silicon (Si) wafers to form molds followed by conformal deposition of parylene C films on the etched Si surface (**Figure** **2**a).^[^ ^54^, ^55^ ^]^ The parylene films are then detached from the wafer. This demolding process results in significant deformation of the parylene nanostructures, causing the average aspect‐ratio to increase from 1.6 to 9.1 (Figure 2b). This is due to parylene's notable plasticity even at room temperature.^[^ ^56^, ^57^ ^]^ The resulting quasi‐random positioning and random heights of the bioinspired parylene nanostructures can be approximated using a Gaussian distribution.^[^ ^32^ ^]^ The parylene nanostructure heights and widths are distributed as *h* = 675 ± 71 nm and *w* = 74 ± 10 nm, respectively, leading to an aspect‐ratio distribution of 9.1 ± 1 (Figure 2c–e) which is very similar to the geometry of the *G. oto* nanostructures (*h* = 650 ± 84 nm, *w* = 73 ± 10 nm, and aspect‐ratio 8.7 ± 1) (Figure S1, Supporting Information). The short‐range pseudo‐periodicity of the parylene nanostructures was measured to be 338 ± 56 nm which was obtained by taking the 2D fast‐Fourier transform (FFT) of the top‐view SEM image. The nanostructures that define the sensing region are fabricated by conformally sputtering Au at high pressures on the same parylene nanostructured films. The optical and sensing nanostructured parylene films were then integrated onto a conventional scleral lens (Figure S2, Supporting Information).

### 2.2 Optical Properties of the Nanostructured Scleral Lens

**Figure 3:**
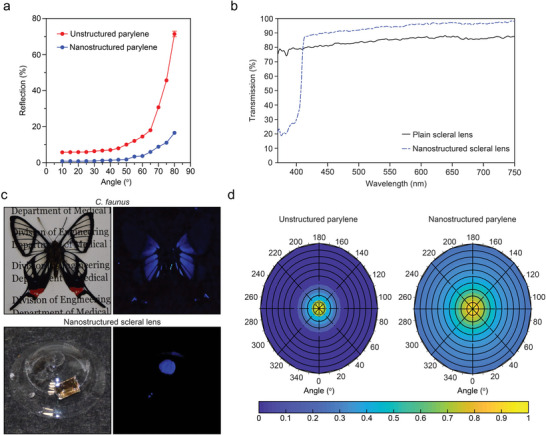
Optical properties of the nanostructured scleral lens. a) Glare‐reduction of nanostructured parylene compared to unstructured parylene at large viewing angles. b) Transmittance of the scleral lens with and without nanostructures under normal incidence. c) Photos of the *C. faunus* butterfly and the nanostructured scleral lens under UV light (365 nm) displaying selective scattering. d) Omnidirectional scattering at 785 nm of Au‐coated parylene nanostructures compared to Au‐coated unstructured parylene.

We characterized the glare‐reduction properties of the bioinspired nanostructures on parylene by measuring the angle‐resolved reflection specifically at large incident angles (10 – 80^o^) where glare effects are most prominent. To ensure a small spot size at large incident angles, we used a custom‐built angle‐resolved reflection spectroscope with an unpolarized 632.8 nm laser source.^[^ ^32^, ^58^ ^]^ The nanostructured parylene displayed a 4.3‐fold lower reflection at 80^o^ (16.6%) compared to a unstructured parylene film (71.5%), which supports the glare‐reduction properties of the nanostructures (**Figure** **3**a, Video S1, Supporting Information). Next, the transmission of nanostructured parylene on the optic zone of the scleral lens was measured at normal incidence (Figure 3b). While a conventional scleral lens (Boston XO) displayed an average VIS (400–750 nm) transmission of 85%, the lens integrated with the parylene nanostructures showed an improved transmission of 93%. Below 400 nm at UVA wavelengths, the average transmission through air for the conventional scleral lens was 78%. The parlyene‐nanostructured lens demonstrated 2.8‐fold lower average transmission (28%) implying a better UVA‐blocking effect. The reduced transmission at UVA can be attributed to the average spacing (≈338 nm) of the high aspect‐ratio parylene nanostructures being comparable to the wavelength of light, thereby leading to increased Mie backscattering. In this case, the pseudo‐periodicity of the nanostructures introduces a phase correlation in the backscattered light, leading to a strong reflection in the UVA regime. Furthermore, the improved transmission in the VIS range can be attributed to the fact that the pseudo‐periodicity of the same nanostructures are now in the subwavelength regime compared to the VIS range resulting in antireflection. This spectral‐zone‐dependent effect has been observed on *C. faunus* wings (Figure 3c).^[^ ^43^ ^]^

Finally, we studied the scattering property of the Au‐coated parylene nanostructures using angle‐resolved NIR reflection spectroscopy. Much like the omnidirectional optical properties of quasi‐random biophotonic nanostructures in nature, omnidirectional scattering could be of great benefit to portable Raman spectroscopy applications where the optical alignment between the sample and the detector is a common issue.^[^ ^50^, ^51^ ^]^ We compared the scattering properties of Au‐coated nanostructured parylene and Au‐coated unstructured parylene at 785 nm which corresponds to the laser wavelength used for our Raman measurements. The angle‐resolved normalized scattering intensity from the unstructured parylene was undetectable beyond 12^o^ due to limitations imposed by the numerical aperture of the collection lens (20x, NA = 24 ^o^) (Figure 3d). In contrast, independent of sample location, the omnidirectional scattering from the Au‐coated parylene nanostructures allowed for continual detection until 36^o^.

### 2.3 Biophysical Properties of the Parylene Nanostructures

**Figure 4:**
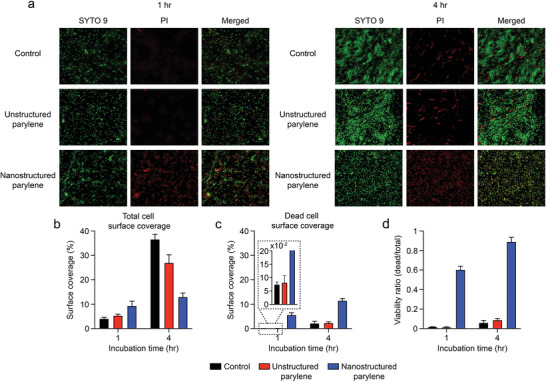
Bactericidal properties of the nanostructured parylene. a) Fluorescent micrographs of a negative control, unstructured parylene, and nanostructured parylene after 1 and 4 h of incubation in a culture of *E. coli*. Adherent bacteria are labeled with cell‐permeable nucleic acid markers SYTO 9 (green) and PI (red). Colocalization of the two stains is shown as merged images. b) Total cell surface coverage, c) dead cell surface coverage, and d) viability ratio measured after 1 and 4 h.

Performing in vitro tests using *Escherichia coli*, we compared the bactericidal behavior of bioinspired nanostructured parylene and unstructured parylene surfaces along with glass slides as negative controls. After incubation times of 1 h and 4 h, bacterial viability was measured using a two‐color fluorescence assay (BacLightTM, ThermoFisher Scientific) consisting of cell‐permeant nucleic stains SYTO 9 and propidium iodide (PI) (**Figure** **4**a). Total surface coverage (measured through area coverage of SYTO 9) by cells was significantly lower (***P < 0.001) on the nanostructured parylene compared to the unstructured parylene and control surfaces (Figure 4b). On average, per field‐of‐view, total surface coverage on the control and unstructured parylene surfaces after 1 h was 4.0% and 5.3%, respectively. After 4 h, the total surface coverage on the two samples was 36.6% and 26.9%, respectively. Furthermore, after 4 h, there was strong evidence of early‐stage biofilm formation on the two samples (Figure S3, Supporting Information). This observation is in agreement with other works that have shown the high susceptibility of unstructured parylene C to *E. coli* colonization.^[^ ^59^, ^60^ ^]^ In comparison, while the nanostructured parylene displayed a marginally higher surface coverage (9.3%) compared to unstructured parylene and control surfaces after 1 h, the total surface coverage by cells after 4 h was considerably impeded (12.9%). The subsequent disruption in growth was due to potent bactericidal activity achieved through a variety of potential means by the nanostructured parylene (see Section S1, Supporting Information). Dead cell surface coverage (measured through area coverage of PI) was significantly higher (***P < 0.001) both after 1 h and 4 h on the nanostructured parylene compared to the control and unstructured parylene surfaces (Figure 4c). As a result, the viability ratio of the bacteria (measured as the ratio of PI:SYTO 9 area coverage) after 4 h was 15‐fold and 10‐fold higher (***P < 0.001) on nanostructured parylene (88.9%) compared to the control (6.0%) and unstructured parylene (8.7%) surfaces, respectively (Figure 4d).

### 2.4 Drop Coating Deposition Raman Spectroscopy on Scleral Lens Sensor

**Figure 5:**
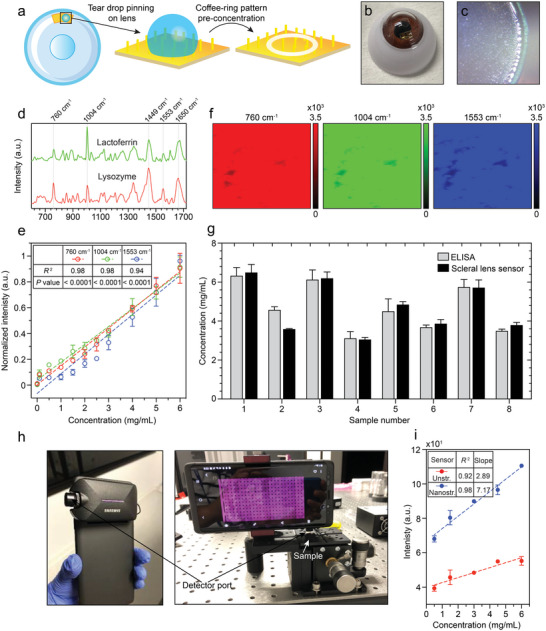
Lysozyme and lactoferrin sensing on nanostructured scleral lens. a) Schematic of DCDRS on the sensing region of the scleral lens. b) Artificial eye with mounted scleral lens. c) Image of the droplet edge showing the coffee‐ring pattern (5x magnification). d) DCDRS spectrum of lactoferrin and lysozyme (concentration: 3 mg mL^−1^) measured independently in PBS. e) Normalized DCDRS intensity of the 760, 1004 and 1553 cm^−1^ peaks of lactoferrin and lysozyme measured together in 1:1 concentrations (0 – 6 mg mL^−1^ each) in artificial tears. f) DCDRS spectrum surface mapping on the droplet edge in a 50 × 50 µm region showing spatial uniformity of the 760, 1004, and 1553 cm^−1^ peaks for artificial tears. g) Calibrated results of the collective concentration of lactoferrin and lysozyme from the scleral lens sensor for 8 different whole tear samples. The results are in good agreement with measurements made using ELISAs. h) Picture of the smartphone integrated with a Raman spectrometer model along with the measurement setup for smartphone measurements. i) Measurement of the 760 cm^−1^ peak intensity of lactoferrin and lysozyme measured together in 1:1 concentrations in PBS using the smartphone detector. The nanostructured sensor shows excellent linearity and improved sensitivity compared to measurements on an unstructured Au parylene film on scleral lens.

We incorporate Au‐coated bioinspired parylene nanostructures on the posterior side of a scleral lens (facing the precorneal tear fluid reservoir) for the label‐free multiplexed detection of lysozyme and lactoferrin using DCDRS (see Section S2, Supporting Information). The proteins are detected within their physiological and pathological concentration ranges of 0 – 6 mg mL^−1^ in tears. The efficient pinning of teardrops on the Au‐coated parylene nanostructures enables localized drying (**Figure** **5**a‐c, Video S2, Supporting Information). The increased hydrophobicity due to the nanostructures over unstructured Au leads to more efficient pre‐concentration of tear proteins resulting in a 10‐fold enhancement of DCDRS signal (Figure S5, Supporting Information).

First, lysozyme and lactoferrin were independently detected in PBS at concentrations ranging from 1–3 mg mL^−1^ (Figure S6, Supporting Information). The raw DCDRS spectra of the two proteins are shown in Figure 5d. Of the various characteristic peaks of the two spectra, the most notable are those at 760, 1004, and 1553 cm^−1^. The 1004 cm^−1^ peak in both spectra along with that of the next most abundant protein in tears, albumin, is primarily due to phenylalanine.^[^ ^48^, ^49^, ^61^, ^62^ ^]^ However, the presence of tryptophan in lysozyme and lactoferrin cause significant differences in their DCDRS spectra compared to albumin and other tear proteins. Lysozyme, which has the highest concentration of tryptophan among the three proteins displays the most prominent peaks at 760 and 1553 cm^−1^.^[^ ^48^, ^49^, ^61^, ^62^ ^]^ Furthermore, due to the unresolved 1014 cm^−1^ mode of tryptophan, the 1004 cm^−1^ peak of lysozyme is slightly broadened and shifted to larger wavenumbers. Lactoferrin, which has the second‐highest tryptophan levels also has peaks at 760 and 1553 cm^−1^ while albumin which has the lowest tryptophan levels does not have discernible peaks in these two regions. As a result, these two peaks specific to lysozyme and lactoferrin can be used to selectively infer their concentrations in whole tears.^[^ ^48^, ^49^, ^61^, ^62^ ^]^

Next, to better simulate the performance of the scleral lens sensor in whole tears, we prepared various concentrations of lysozyme and lactoferrin between 0 and 6 mg mL^−1^ in an artificial tear buffer consisting of other tear proteins such as albumin and immunoglobulins as well as electrolytes that maintain osmolarity such as Na^+^, K^+^, Cl^−^, and HCO_3_^−^ (see Experimental Methods Section). The concentrations of lysozyme and lactoferrin were varied in a 1:1 ratio in all cases to aid the simplicity of measurements. This is because a collective and marked lowering of both lysozyme and lactoferrin concentrations have been observed in dry eye disease patients over healthy subjects.^[^ ^63^, ^64^, ^65^ ^]^ Consequently, their concentrations can be studied in conjunction for this specific application. However, to obtain a finer resolution of measurements at lower concentrations, 1:1 increments of 0.5 mg mL^−1^ were used between 0 and 3 mg mL^−1^ while increments of 1 mg mL^−1^ were used between 3 and 6 mg mL^−1^. The 760, 1004, and 1553 cm^−1^ peaks were tracked in each case with excellent linearity being observed (Figure 5e). In addition, Raman surface mapping performed on the scleral lens over a 50 × 50 µm area on the dried droplet edge at the three peak locations indicates spatial uniformity of the DCDRS approach (Figure 5f).

The nanostructured scleral lens was then employed to detect lysozyme and lactoferrin concentration in eight different whole human tear samples obtained from healthy subjects. The measurements taken using the artificial tear buffer were used as characteristic curves to map the collective concentrations of lysozyme and lactoferrin in whole tears. For reference measurements, the lactoferrin and lysozyme concentrations in the same tear samples were detected in conjunction using commercial enzyme‐linked immunosorbent assays (ELISAs). To minimize the effects of potential differences in the constitution of the artificial tear buffer and whole tears, the measurements from the scleral lens sensor were calibrated against those from the ELISAs (see Supplementary Section S3, Supporting Information). Post‐calibration, the average difference between the means of corresponding measurements for a given tear sample taken from the scleral lens sensor and the ELISAs (performed in triplicate) was 0.27 mg mL^−1^ (Figure 5g). Furthermore, the standard deviation (SD) of measurements taken on the scleral lens sensor for a given tear sample was 0.22 mg mL^−1^. In comparison, the SD for the ELISA on average was slightly higher (0.33 mg mL^−1^). The lower SD on average for the measurements from the scleral lens sensor compared to the ELISA could be attributed to the inherent simplicity (single‐step vs multi‐step) and assay time (30 min vs 8 h) of our approach that greatly minimizes user error.

Finally, the suitability of the nanostructured scleral lens to point‐of‐care self‐monitoring applications was validated by performing DCDRS signal detection using a custom‐built Raman spectrometer module integrated onto a smartphone (Figure 5h). The frequently reported issue when using handheld/portable Raman solutions pertains to sample‐detector alignment.^[^ ^50^, ^51^ ^]^ As a feasibility demonstration, Figure 5i shows the collective DCDRS spectrum of lysozyme and lactoferrin detected on the nanostructured scleral lens at a 1:1 ratio for concentrations ranging between 0 and 6 mg mL^−1^ in PBS. Here, the intensity of the 760 cm^−1^ peak was specifically tracked as it was covered by the spectral range of the smartphone detector. For comparison, results for the same concentration levels for an unstructured Au parylene film mounted on the lens is also shown. Here, the nanostructured scleral lens demonstrated excellent linearity (*R* ^2^ = 0.98) and was ≈2.5 times more sensitive (slope = 7.17) than the unstructured Au parylene film on the scleral lens (slope = 2.89).

## 3 Conclusion

Inspired by the quasi‐random nanostructures on the wings of the *G. oto* butterfly, we created a multifunctional bioinspired nanostructured scleral lens for dry eye disease monitoring. The parylene nanostructures on the lens offer glare‐reduction: the lens reflected as little as 16.6% of incident light at large viewing angles up to 80^o^ and thereby mitigated a common issue faced by contact lens wearers. Furthermore, the nanostructures reduce UVA transmission by 2.8‐fold compared to a plain scleral lens while simultaneously improving transmission in the visible regime by 1.1‐fold. Beyond optical properties, the parylene nanostructures display potent bactericidal activity against *E. coli* studied in vitro killing 10‐fold more bacteria compared to unstructured parylene within 4 h. Finally, the same nanostructures are conformally coated with Au to form a sensing region on the posterior side of the scleral lens away from the optic zone. This sensing region is used to detect two tear protein biomarkers of dry eye disease – lysozyme and lactoferrin using label‐free DCDRS. The two proteins were first detected within their physiological and pathological concentration range in an artificial tear buffer. These results were then used to map concentration levels in whole human tears from multiple subjects. The measurements using the nanostructured scleral lens sensor were in good agreement with those taken concurrently using ELISAs. In addition, protein concentration measurements were also performed using a custom‐built Raman spectrometer module integrated into a smartphone, proving this approach suitable for potential point‐of‐care self‐monitoring applications. With the strong need for tear biomarker analyses to better understand the onset and progression of various ocular pathologies, we envisage that such a multifunctional scleral lens sensor coupled with smartphone‐based monitoring can be used to better inform clinicians and patients alike.

## 4 Experimental Section

### High‐Resolution Imaging

Dried wings of *G. oto* were purchased from Bicbugs LLC (Arizona, USA). The wing samples were coated with a 15 nm Au layer (Lesker Labline E‐beam Evaporator, Kurt J. Lesker) before examination by SEM (FEI Nova 200 NanoLab Dualbeam) operated at 5 kV. The parylene nanostructures were conformally coated with a 20 nm Au layer (ATC Orion 8, AJA International Inc., USA) for imaging.

### Surface Characterization

ImageJ (https://imagej.nih.gov/ij/), a public domain and Java‐based image processing tool, was used for statistical analysis of nanostructure size on the parylene nanostructure samples. The 2D Fourier power spectra were obtained from SEM images and calculated with a fast Fourier transform algorithm in MATLAB.

### Bioinspired Nanostructured Parylene Fabrication and Scleral Lens Integration

Positively‐charged polyelectrolyte poly(diallyldimethylammonium) (PDDA, Sigma Aldrich, USA) was first pipetted onto the surface of an Si wafer at a concentration of 0.2% in DI water. The solution was allowed to sit on the Si surface for 10 min following which the wafer was dried under a stream of N_2_. Next, a colloidal solution of negatively‐charged polystyrene nanoparticles (PS NPs, diameter: 200 nm, Sigma Aldrich, USA) at a concentration of 0.2% in ethanol was pipetted and spin‐coated (300 rpm for 10 s followed by 500 rpm for 2 min) on the surface. The coated Si surface was then exposed to O_2_ plasma (Plasmalab System 100 ICP‐RIE 380, Oxford Instruments, United Kingdom) to shrink the diameters of the PS NP to 75 nm. Next, a 15 nm thick Al_2_O_3_ hard mask was directionally evaporated using E‐beam evaporation (CHA Mark 40, CHA Industries, USA) at a rate of 0.5 Å s^−1^. The PS NPs were then removed through tape‐stripping using dicing tape. Next, a Bosch process was performed with the hard mask to etch nanoholes into the Si surface (Plasmalab System 100 ICP‐RIE 380, Oxford Instruments, United Kingdom). Following hard mask removal in BOE, 20 µm thick parylene C films were conformally deposited over the etched Si surface (LabTop 3000, Para Tech, USA). The parylene films were then demolded from the wafer by gently peeling them. To create the Au‐coated sensing region, the nanostructures were conformally coated with 60 nm‐thick Au through sputtering (ATC Orion 8, AJA International Inc., USA) at a rate of 0.2 Å s^−1^. Sections of clear and Au‐coated nanostructured parylene films were then mounted onto the anterior and posterior side of a fixtured scleral lens. First, a small amount of optically‐clear UV curable epoxy (Loctite 3105, Henkel AG & Company, KGaA, Germany) was gently applied onto the lens surface using a pipette tip. Next, the parylene films were placed on top of the coated lens and held in place using forceps to allow for the epoxy to evenly spread between the two surfaces. The parylene films were then flash cured in situ using a broadband UV lamp for 1 min followed by a longer 30 min curing step to create a permanent bond. The size of the films (diameter: 5 mm) were maintained to be considerably smaller than that of the scleral lens to preserve oxygen permeability.

### Optical Spectroscopic Analyses

Angle‐resolved VIS reflection measurements of the samples were made using a custom‐built optical goniometric setup. A stabilized He‐Ne laser (632.8 nm) was used to measure reflection up to 80° for the glare measurement and the reflectance was measured through a fiber‐coupled USB spectrometer (AvaSpec‐ULS2048CL‐EVO, Avantes). The aperture angle was set to 3°. The optical fiber and the sample holder were rotatable in the *x–y* plane to measure the specular angle‐resolved reflectance spectra.

Transmission properties of the lens with and without parylene nanostructures were measured using a customized optical microscope operating in bright‐field (BF) mode. A halogen lamp was employed as a light source using an abbe condenser with a numerical aperture of (NA) = 0.9. The transmitted light was collected in a confocal configuration using a 50 µm core optical fiber and analyzed using a spectrometer (AvaSpec‐ULS2048CL‐EVO, Avantes). A stabilized tungsten–halogen light source (SLS201, ThorLabs) was collimated to form a 500 µm wide parallel incident beam that illuminates the sample at a normal incident.

Omnidirectional scattering of the Au‐parylene structures was measured by placing samples on a stage that allows for tilt and rotation. The samples were probed by a ×20 objective lens (NA = 0.42) interfaced with an NIR light source (SLS201, ThorLabs), a mini‐spectrometer (Maya200 Pro, Ocean Optics), and a CCD camera (ThorLabs).

The subsequent data analysis was done with MATLAB together with the commercial software packages from the spectrometer. All measurements were taken in the dark to avoid possible stray light from the surrounding. A reference measurement was performed with a mirror and a calibrated commercial spectralon to calculate the relative specular and diffuse (scattering) reflectance, respectively.

### Bactericidal Assay

A culture of *E. coli* (DH5 Alpha competent cells, Zymo Research, USA) was inoculated in fresh lysogeny broth (LB) overnight at 37 °C under gentle shaking (250 rpm). On recovery, the bacteria were diluted in fresh LB to an OD_600_ of 0.25. Next, negative cover glass control, unstructured parylene, and nanostructured parylene surfaces were incubated in 2 mL of the bacterial culture. After 1 h and 4 h incubations, the surfaces with adherent bacteria were gently washed with and incubated in 0.85% NaCl solution to remove traces of growth medium. Following this, the surfaces were stained with a commercial bacterial viability kit following the manufacturer's protocols (LIVE/DEAD BacLight Bacterial Viability Kit, ThermoFisher Scientific, USA). Fluorescence images were captured from the surfaces using a wide‐field epifluorescence microscope (20x objective, Leica DMI 600, Leica AG). ImageJ was used to perform thresholding and bacterial surface coverage calculations on five independent fields of view per sample.

### Artificial Tear Buffer Preparation and Whole Tear Collection

Artificial tear buffer was prepared using previously reported methods.^[^ ^66^, ^67^ ^]^ Briefly, electrolytes K^+^, HCO_3_^−^, Na^+^, and Cl^−^ were added to DI water at concentrations of 24, 24, 130, and 130 mM, respectively. Furthermore, uric acid at a concentration of 75 µM was added. Following this, representative tear proteins such as albumin and IgG were added at concentrations of 0.02 mg mL^−1^ and 3 µg mL^−1^, respectively. Finally, various concentrations of lysozyme and lactoferrin ranging from 0 to 6 mg mL^−1^ each were added to the prepared tear buffer in a 1:1 ratio.

While basal tears are arguably more representative of the precorneal tear fluid reservoir, their collection requires specialized equipment such as microcapillary tubes and careful prior stimulation with different volumes of saline into the cul‐de‐sac, and as a result, considerable subject compliance.^[^ ^68^ ^]^ Instead, as a proof‐of‐concept, reflex tears in response to a gentle stream of air were collected from consenting healthy volunteers. No ethics board/committee approval was required as no animal testing and no invasive sample collection from humans was performed.

### Raman Spectrometer Module on Smartphone

A separate optical module developed in‐house was connected to a smartphone to measure the Raman signal through the smartphone. The optical module consists of a laser diode of 785 nm, a series of optics to collect and direct the scattered light toward the detector, and a notch filter to block the direct Rayleigh scattering at 785 nm. The on‐chip CMOS image sensor in the smartphone was used as the detector. To measure the spectral response, a series of 128 resonant distributed Bragg filters was embedded on top of the image sensor. The filter transmission resonances were varied from 806.5 nm to 880.45 nm to measure the Raman scattering in the spectrum range of 339.6 cm^−1^–1381.03 cm^−1^. The subsequent data and image analysis was done with MATLAB to extract the scattering intensity in the target spectrum range. More details of the Raman module can be found in these disclosures.^[^ ^69^, ^70^, ^71^ ^]^

### Statistics

Optical glare measurements were performed in *n* = 4 sample locations. Results from the bacterial assay were processed using *n* = 5 independent fields‐of‐view images per sample. A two‐way analysis of variance was used to determine the statistical significance of the difference between samples using Prism GraphPad 8 software. A *P*‐value < 0.05 was considered statistically significant. Tear protein measurements were performed *n* = 3 times. All data with error bars were represented as mean ± standard deviation.

## Conflict of Interest

The authors declare no conflict of interest.

## Supporting information

Supporting Information

Supplemental Video 1

Supplemental Video 2

## Data Availability

The data that support the findings of this study are available on request from the corresponding author. The data are not publicly available due to privacy or ethical restrictions.
