# Supplementary material for: Glasswing‐Butterfly‐Inspired Multifunctional Scleral Lens and Smartphone Raman Spectrometer for Point‐of‐Care Tear Biomarker Analysis
Source: Adv Sci (Weinh). 2022 Dec 1;10(5):2205113. doi: 10.1002/advs.202205113 (PMC9929119; doi:10.1002/advs.202205113)
Supplement: Supplementary file 1 — Supporting Information [file ADVS-10-2205113-s002.pdf]

ADVANCED SCIENCE
Open Access

Supporting Information

for *Adv. Sci.*, DOI 10.1002/advs.202205113

Glasswing-Butterfly-Inspired Multifunctional Scleral Lens and Smartphone Raman Spectrometer for Point-of-Care Tear Biomarker Analysis

Vinayak Narasimhan, Radwanul Hasan Siddique, Un Jeong Kim, Suyeon Lee, Hyochul Kim, YoungGeun Roh, Yibing Michelle Wang and Hyuck Choo**

Supporting Information

Glasswing-butterfly-inspired multifunctional scleral lens and smartphone Raman spectrometer for point-of-care tear biomarker analysis

Vinayak Narasimhan, Radwanul Hasan Siddique, Un Jeong Kim, Suyeon Lee, Hyochul Kim, YoungGeun Roh, Yibing Michelle Wang, and Hyuck Choo**

Section S1: Bactericidal effect of the parylene nanostrutures

The total free energy change of a bacterial cell adhering to a nanostructured surface can be represented as a function of energetic contributions from stretching and bending of the bacteria due to the nanostructures as well as the process of adhesion itself. Mathematically, this can be expressed as,[1,2]

$$\Delta E = \frac{1}{2}\lambda\frac{\Delta S^2}{S_o} + \int_{S_{cell}} [\frac{\kappa}{2}(c_1 + c_2 - c_o)^2]dA - \int_{S_{ad}} \gamma\, dA - E_o^{bend}$$

where λ is the stretching modulus of the cell membrane, ΔS and S_o are the total surface area change and initial surface area of the cell, κ is the bending modulus of the cell membrane, c_1, c_2 and c_o are the two principal curvatures and the spontaneous curvature of the bending membrane, γ is the surface energy density between the cell membrane and the nanostructured surface. In the above expression, the first term represents the energy contribution due to stretching of the overall bacterial surface area. The second energy contribution due to bending, integrated over the entire surface area of the cell, can be expressed using the Canham-Helfrich Hamiltonian.[3,4] The third term corresponds to the energy released due to the process of adhesion of the cell onto the nanostructured surface while the fourth term is the bending energy due to the deformed shape of the cell membrane in free space. As shown in prior work,[1,2] the total free energy change can be represented purely as a function of two variables:

the half-width r and the contact angle θ of the bacterial cell on the nanostructured surface. This is illustrated Figure S4a schematically for a rod-shaped bacterium like *Escherichia coli* on quasi-random nanostructures. As a result, minimization of the total free energy change can be performed as a function of (r,θ).

The nominal values of various constants in the free energy change minimization process can be inferred from literature. For *E. coli*, $\lambda \approx 0.25 k_B T$ nm^{-2},[5] $\kappa \approx 10 k_B T$ [6,7] and $\gamma \approx 0.2 k_B T$ nm^{-2},[8] where $k_B T \approx 4.11 \times 10^{-21}$ J. As a result, for a given nanostructured surface geometry (i.e. nanopillar width and short-range periodicity), the minimization can be performed to obtain the equilibrium (r,θ) pairing as shown in Figure S4b.

This analysis can be performed for a range of nanostructured surface geometries (i.e. varying nanopillar widths and short-range periodicities). For every geometric condition, the minimization can be iteratively performed. As a result, in the equilibrium stage of the adhesion process, parameters such as the stretching degree ($\frac{\Delta S}{S_o}$) and the energy contributions due to stretching ($E_{stretch} = \frac{1}{2}\lambda\frac{\Delta S^2}{S_o}$), bending ($E_{bend} = \int_{S_{cell}}[\frac{\kappa}{2}(c_1 + c_2 - c_o)^2]dA$) and adhesion ($E_{ad} = \int_{S_{ad}} \gamma \, dA$) can be extracted. The stretching degree for various geometric conditions is shown in Figure S4c. The stretching degree computed for the parylene nanostructures given their average half-width and short-range periodicity ($\frac{\Delta S}{S_o} \approx 0.18$) is comparable to results obtained by other thermodynamic models.[2,9,10] While this level of stretching degree may not result in rupturing of the bacterial cell membrane, such a deformation may be sufficient in producing alterations to the genomic and proteomic profile of adherent bacteria.[11–13] This could potentially result in programmed or systemic cell death (as opposed to physical lysis) through pathways such as the induction oxidative stress[11,14,15] and various cell signaling mechanisms.[11,13,16,17]

In a similar fashion, $E_{stretch}$, E_{bend} and E_{ad} can be computed. $|E_{stretch}| + |E_{bend}| < |E_{ad}|$ presents a thermodynamically favorable condition for bacterial adhesion on the nanostructured surface as the free energy of the entire system can be further lowered due to the adhesion process. In the same vein, $|E_{stretch}| + |E_{bend}| > |E_{ad}|$ presents a thermodynamically unfavorable condition for bacterial adhesion. This energy difference (i.e. $|E_{stretch}| + |E_{bend}| - |E_{ad}|$), computed at the equilibrium condition for different R_p and D_p is shown in Figure S4d. In our regime of interest, the condition $|E_{stretch}| + |E_{bend}| < |E_{ad}|$ always holds ensuring an increased adhesion of *E. coli* on the parylene nanostructured surface as seen after 1 h of incubation experimentally (Figure 4b).

Much like observations on cicada[9,18] and dragonfly[19,20] wings, large surface roughness due to the presence of nanopillars display high initial adhesion of high-motility bacteria such as *E.coli*.[2] Upon adhesion, the nanopillars increase the deformation energy of the cell membrane.[1,2,20] Gram-negative bacterial membrane envelopes as in the case of *E. coli* are thin (~ 5 nm) and undergo larger deformations due to low bending and stretching moduli.[1,2] Consequently, bactericidal effects have been reported to occur through various modes – the most notable of which being mechanical rupture or lysis.[1,2,9,21] However, recent work has revealed that the occurrence and subsequent influence of lysis (or loss of cytosolic content) is negligible.[11] Instead, the deformations, while not leading to physical lysis, cause alterations to the genomic and proteomic profile of the bacteria.[11–13] Proteomics performed on *E. coli* populations whose growth was greatly impaired by nanostructures revealed numerous differentially expressed proteins (DEPs) largely associated with protection from oxidative stress,[11,14,15] irreversible protein aggregation,[11] DNA protection and membrane stress response.[13] As a result, significantly increased levels reactive oxygen species (ROS) like H_2O_2 that mediate damage of DNA, lipids and proteins lead to cell death.[11,16,17] This explains the killing rates of various naturally-occurring and synthetic nanostructures like our nanostructured parylene within short timespans (~1 – 4 hr).[11,19,20]

Section S2: DCDRS of lysozyme and lactoferrin in human tears

The origination of contact angle hysteresis on geometrically heterogenous surfaces is a well understood concept.[22–24] Hysteresis acts as an energy barrier to droplet movement on rough surfaces leading to droplet pinning.[25–27] Contact angle hysteresis is shown to have a proportional relationship to work of adhesion (or pinning force per unit length) which is the work done to separate the two adjacent liquid-liquid and liquid-solid phases.[27,28] The evaporation at the edge of the pinned droplet, along with capillary forces that transport material from the droplet bulk to the edge leads to the formation of the so-called coffee-ring pattern.[29,30] Such an approach has been used to segregate, pre-concentrate, and detect through Raman spectroscopy, various solutes in complex biological fluids.[31–34] Of note, this approach has been particularly useful for the proteomics of human tears which has a total protein concentration (TPC) approximately 7-fold lower than blood serum.[32,35] Studies have also shown that the same process causes the segregation of different solutes, for instance, salts and proteins in tears, with salts crystalizing closer to the center of the dried drop and proteins being deposited at the edge.[31,36] The detection method, known as drop coating deposition Raman spectroscopy (DCDRS) has been validated to produce highly reproducible Raman spectra from different drops, droplet volumes, protein concentrations, and substrates.[37] Preliminary DCDRS approaches have only used highly reflective, non-wetting substrates with minimal Raman background such as atomically flat Au.[31,38] However, recent work has shown that the introduction of nanostructures to DCDRS substrates which cause increased hydrophobicity and droplet pinning along with smaller drying patterns leads to more efficient solute pre-concentration and as a result, an enhancement of DCDRS signal.[39] Furthermore, unlike SERS, DCDRS has been reported to show fantastic correlation with the Raman spectra of lyophilized tear proteins.[31,34] In other words, the origin of peaks in DCDRS signals for tears can be easily assigned to specific constituent amino acids.[31,34] SERS studies on tears

however, have shown that the origin of the SERS signal may be from non-protein constituents.[34]

While tears have been studied to contain hundreds of different proteins,[40] ~99% of the TPC is made up of lysozyme, lactoferrin and tear-specific prealbumin.[31] Furthermore, typical concentrations of lysozyme and lactoferrin in tears (2 – 3 mg/mL) are an order of magnitude greater than albumin (~0.2 mg/mL)[31,41] making them potentially easy to assay. Lysozyme and lactoferrin have been most notably shown to be biomarkers of chronic dry eye disease with patients exhibiting significantly lower concentrations of two proteins in tears relative to healthy subjects.[42–44] Despite the ever-increasing prevalence of dry eye disease, current diagnostic tests that primarily assess the ocular surface and tear film production levels are often inconclusive on their own, requiring the need for tear biomarker analyses to aid accurate diagnosis.[45–47] While various commercially available techniques for tear biomarker analyses exist, they are often expensive and require specialized equipment, operators, and reagents.[46,48]

In this regard, CL-based sensors offer a simple, personalized, and point-of-care self-monitoring approach to dry eye disease diagnostics. Amongst the various sensing modalities available on CL-based sensors (Table S1), an approach relying on passive nanophotonic optical components is simple and cost-effective. It requires no power supply or data transmission circuitry making it amenable for point-of-care applications.[49] Furthermore, scleral lenses are better suited for and often prescribed to dry eye disease patients over traditional soft contact lenses.[49,50] As a result, optical components integrated onto scleral lenses for dry eye disease monitoring through the detection of tear biomarkers may hold great promise.

Section S3: Scleral lens sensor calibration

To account for potential differences between the constituent profile of artificial and whole tears, the results from the scleral lens sensor for whole tears was calibrated those taken using two individual commercial enzyme-linked immunosorbent assays (ELISAs) with absolute human lysozyme and lactoferrin standards.[51,52] First, the DCDRS results for lysozyme and lactoferrin in artificial tears as shown in Figure 5e were used as characteristic curves to map the DCDRS spectra in whole tears to collective lysozyme and lactoferrin concentration levels (Figure S7a). However, compared to the collective concentration of the two proteins measured independently using ELISAs, the corresponding measurement from the scleral lens sensor was consistently lower. This could be attributed to greater complexity of whole tears compared to artificial tears as well as differences in the concentration profiles of lysozyme and lactoferrin in basal (after which the artificial tears were modeled) and reflex (tear samples collected) tears. Studies have indicated greater lysozyme and lactoferrin concentrations in reflex tears compared to basal tears which could explain the lower measurements from the scleral lens sensor.[53]

The aggregated concentration of lysozyme and lactoferrin from the independent ELISA measurements was computed as,

$$\mu_{agg} = \mu_{lys} + \mu_{lact}$$

$$\sigma_{agg} = \sqrt{\sigma_{lys}^2 + \sigma_{lact}^2}$$

where μ_{lys} and μ_{lact} correspond to the means of the independently measured concentrations of lysozyme and lactoferrin while σ_{lys} and σ_{lact} are their standard deviations (SDs), respectively. Comparing the aggregated means of the ELISA measurements with means of scleral lens sensor measurements, the difference on average for a given tear sample was 0.93 mg/mL (Figure S7b). Calibration of the scleral lens measurements is performed by adding this average difference to the uncalibrated results as shown in Figure 5g.

Section S4: Nanostructure stability on scleral lens

Given the nanostructures are of high aspect-ratio and made of a polymer, they are understandably sensitive to touch or direct hard contact. However, due to the innate plasticity of parylene, we expect the nanostructures to be more robust and indeed we did not see signs of degradation after three months. Moreover, this is further remedied by considering how scleral lenses are to be handled, inserted, positioned, and removed. It is recommended that scleral lenses be aseptically inserted and removed by using plungers with most users preferring this approach.[54] The usage of plungers can ensure handling without contacting the nanostructures.

When the scleral lens is worn, it interacts with the tear film of the eye. Traditional scleral lenses are typically hydrophilic to enable good wetting thereby creating a pre-lens (anterior) and post-lens (posterior) tear film.[55] While various studies estimate different thickness ranges for these tear films, they are consistently reported to be at least several microns in thickness (3 – 40 um).[56–59] This thickness is considerably larger than the nanostructure thickness which is submicron. As a result, both the anterior and posterior side nanostructures would be fully enveloped by the pre- and post-lens tear films, respectively. This would ensure no disruption to their morphology during use. The blinking of the eyelid in this case would replenish the pre-lens tear film thereby continuing to preserve the nanostructures. In addition, commercially available lubricating eye drops can be used to ensure adequate hydration.

Other factors to consider are oxygen permeability and water content. Parylene is often used as a permeation barrier for medical devices due to its low oxygen permeability and water vapor transmission rate. In contrast, scleral lenses are designed to be gas permeable which enables transmission of oxygen to the eyes.[60] However, they are not designed to designed to hold water like soft contact lenses and as a result, do not dehydrate as easily. Owing to this, scleral lenses are often prescribed for dry eye disease.[61,62] Given this mismatch in gas permeability

between parylene and traditional scleral lenses, we chose to design the nanostructured parylene film to be considerably smaller than the scleral lens. For instance, the optical/bactericidal nanostructured parylene film mounted on the anterior side of the scleral lens has a diameter of 5 mm which is comparable to the average pupil size in adults. Compared to this, modern scleral lenses can be as large as 25 mm in diameter having a 25-fold larger surface area thereby preserving the oxygen permeability of a lens that is nanostructured.[60]

References

[1] X. Li, *Phys. Chem. Chem. Phys.* **2016**, *18*, 1311.

[2] X. Li, T. Chen, *Phys. Rev. E* **2016**, *93*, 52419.

[3] P. B. Canham, *J. Theor. Biol.* **1970**, *26*, 61.

[4] W. Helfrich, *Zeitschrift für Naturforsch. C* **1973**, *28*, 693.

[5] Y. Sun, T.-L. Sun, H. W. Huang, *Biophys. J.* **2014**, *107*, 2082.

[6] D. Marsh, *Chem. Phys. Lipids* **2006**, *144*, 146.

[7] E. N. Cytrynbaum, Y. D. Li, J. F. Allard, H. Mehrabian, *Phys. Rev. E* **2012**, *85*, 11902.

[8] X. Zhang, Q. Zhang, T. Yan, Z. Jiang, X. Zhang, Y. Y. Zuo, *Environ. Sci. Technol.* **2015**, *49*, 6164.

[9] S. Pogodin, J. Hasan, V. A. Baulin, H. K. Webb, V. K. Truong, T. H. Phong Nguyen, V. Boshkovikj, C. J. Fluke, G. S. Watson, J. A. Watson, R. J. Crawford, E. P. Ivanova, *Biophys. J.* **2013**, *104*, 835.

[10] S. Wu, F. Zuber, K. Maniura-Weber, J. Brugger, Q. Ren, *J. Nanobiotechnology* **2018**, *16*, 20.

[11] J. Jenkins, J. Mantell, C. Neal, A. Gholinia, P. Verkade, A. H. Nobbs, B. Su, *Nat. Commun.* **2020**, *11*, 1626.

[12] L. Rizzello, A. Galeone, G. Vecchio, V. Brunetti, S. Sabella, P. P. Pompa, *Nanoscale Res. Lett.* **2012**, *7*, 575.

[13] L. Rizzello, B. Sorce, S. Sabella, G. Vecchio, A. Galeone, V. Brunetti, R. Cingolani, P. P. Pompa, *ACS Nano* **2011**, *5*, 1865.

[14] S. Kang, M. Herzberg, D. F. Rodrigues, M. Elimelech, *Langmuir* **2008**, *24*, 6409.

[15] M. Olivi, E. Zanni, G. De Bellis, C. Talora, M. S. Sarto, C. Palleschi, E. Flahaut, M. Monthioux, S. Rapino, D. Uccelletti, S. Fiorito, *Nanoscale* **2013**, *5*, 9023.

[16] L. C. N. da Silva, in (Ed.: R.C. Diniz), IntechOpen, Rijeka, **2017**, p. Ch. 6.

[17] Y. Hong, J. Zeng, X. Wang, K. Drlica, X. Zhao, *Proc. Natl. Acad. Sci.* **2019**, *116*, 10064 LP.

[18] J. Hasan, H. K. Webb, V. K. Truong, S. Pogodin, V. A. Baulin, G. S. Watson, J. A. Watson, R. J. Crawford, E. P. Ivanova, *Appl. Microbiol. Biotechnol.* **2013**, *97*, 9257.

[19] E. P. Ivanova, J. Hasan, H. K. Webb, G. Gervinskas, S. Juodkazis, V. K. Truong, A. H. F. Wu, R. N. Lamb, V. A. Baulin, G. S. Watson, J. A. Watson, D. E. Mainwaring, R. J. Crawford, *Nat. Commun.* **2013**, *4*, 2838.

[20] C. D. Bandara, S. Singh, I. O. Afara, A. Wolff, T. Tesfamichael, K. Ostrikov, A. Oloyede, *ACS Appl. Mater. Interfaces* **2017**, *9*, 6746.

[21] F. Xue, J. Liu, L. Guo, L. Zhang, Q. Li, *J. Theor. Biol.* **2015**, *385*, 1.

[22] D. Öner, T. J. McCarthy, *Langmuir* **2000**, *16*, 7777.

[23] L. Gao, T. J. McCarthy, *Langmuir* **2006**, *22*, 6234.

[24] L. Gao, T. J. McCarthy, *Langmuir* **2006**, *22*, 2966.

[25] C. G. L. Furmidge, *J. Colloid Sci.* **1962**, *17*, 309.

[26] A. Al-Sharafi, B. S. Yilbas, H. Ali, N. AlAqeeli, *Sci. Rep.* **2018**, *8*, 3061.

[27] Y. Xiu, L. Zhu, D. W. Hess, C. P. Wong, *J. Phys. Chem. C* **2008**, *112*, 11403.

[28] B. M. L. Koch, A. Amirfazli, J. A. W. Elliott, *J. Phys. Chem. C* **2014**, *118*, 18554.

[29] R. D. Deegan, O. Bakajin, T. F. Dupont, G. Huber, S. R. Nagel, T. A. Witten, *Nature* **1997**, *389*, 827.

[30] S. Armstrong, G. McHale, R. Ledesma-Aguilar, G. G. Wells, *Langmuir* **2019**, *35*, 2989.

[31] J. Filik, N. Stone, *Analyst* **2007**, *132*, 544.

[32] J. Filik, N. Stone, *Anal. Chim. Acta* **2008**, *616*, 177.

[33] J. Filik, N. Stone, *J. Raman Spectrosc.* **2009**, *40*, 218.

[34] P. Hu, X.-S. Zheng, C. Zong, M.-H. Li, L.-Y. Zhang, W. Li, B. Ren, *J. Raman Spectrosc.* **2014**, *45*, 565.

[35] V. Ng, P. Cho, *Graefe's Arch. Clin. Exp. Ophthalmol.* **2000**, *238*, 571.

[36] D. Zhang, M. F. Mrozek, Y. Xie, D. Ben-Amotz, *Appl. Spectrosc.* **2004**, *58*, 929.

[37] C. Ortiz, D. Zhang, Y. Xie, A. E. Ribbe, D. Ben-Amotz, *Anal. Biochem.* **2006**, *353,* 157.

[38] D. Zhang, Y. Xie, M. F. Mrozek, C. Ortiz, V. J. Davisson, D. Ben-Amotz, *Anal. Chem.* **2003**, *75*, 5703.

[39] E. Kočišová, M. Petr, H. Šípová, O. Kylián, M. Procházka, *Phys. Chem. Chem. Phys.* **2017**, *19*, 388.

[40] G. A. de Souza, L. M. F. de Godoy, M. Mann, *Genome Biol.* **2006**, *7*, R72.

[41] V. Ng, P. Cho, C. To, *Graefe's Arch. Clin. Exp. Ophthalmol.* **2000**, *238*, 738.

[42] P. T. Janssen, O. P. van Bijsterveld, *Exp. Eye Res.* **1983**, *36*, 773.

[43] Y. Ohashi, R. Ishida, T. Kojima, E. Goto, Y. Matsumoto, K. Watanabe, N. Ishida, K. Nakata, T. Takeuchi, K. Tsubota, *Am. J. Ophthalmol.* **2003**, *136*, 291.

[44] G. Hernández-Molina, T. Sánchez-Hernández, *Semin. Arthritis Rheum.* **2013**, *42*, 627.

[45] S. P. Phadatare, M. Momin, P. Nighojkar, S. Askarkar, K. K. Singh, *Adv. Pharm.* **2015**, *2015*, 704946.

[46] S. D'Souza, L. Tong, *Eye Vis.* **2014**, *1*, 6.

[47] N. S. Roy, Y. Wei, E. Kuklinski, P. A. Asbell, *Invest. Ophthalmol. Vis. Sci.* **2017**, *58*, BIO1.

[48] H. R. Culver, M. E. Wechsler, N. A. Peppas, *ACS Nano* **2018**, *12*, 9342.

[49] A. K. Yetisen, N. Jiang, C. M. Castaneda Gonzalez, Z. I. Erenoglu, J. Dong, X. Dong, S. Stößer, M. Brischwein, H. Butt, M. F. Cordeiro, M. Jakobi, O. Hayden, A. W. Koch, *Adv. Mater.* **2020**, *32*, 1906762.

[50] D. S. Jacobs, P. Rosenthal, *Cornea* **2007**, *26*.

[51] "Human Lysozyme ELISA Kit," can be found under https://www.abcam.com/human-lysozyme-elisa-kit-lzm-ab108880.html, **n.d.**

[52] "Human Lactoferrin ELISA Kit," can be found under https://www.abcam.com/human-lactoferrin-elisa-kit-hlf2-ab108882.html, **n.d.**

[53] R. N. Stuchell, R. L. Farris, I. D. Mandel, *Ophthalmology* **1981**, *88*, 858.

[54] R. J. Macedo-de-Araújo, E. van der Worp, J. M. González-Méijome, *Contact Lens Anterior Eye* **2020**, *43*, 553.

[55] M. Serramito, A. Privado-Aroco, L. Batres, G. Carracedo G, *Contact Lens Anterior Eye* **2019**, *42*, 520.

[56] M. J. Glasson, F. Stapleton, L. Keay, M. D. P. Willcox, *Contact Lens Anterior Eye* **2006**, *29*, 41.

[57] J. I. Prydal, P. Artal, H. Woon, F. W. Campbell, *Invest. Ophthalmol. Vis. Sci.* **1992**, *33*, 2006.

[58] J. Wang, D. Fonn, T. L. Simpson, L. Jones, *Invest. Ophthalmol. Vis. Sci.* **2003**, *44*, 2524.

[59] P. E. KING-SMITH, B. A. FINK, N. FOGT, *Optom. Vis. Sci.* **1999**, *76*.

[60] E. van der Worp, D. Bornman, D. L. Ferreira, M. Faria-Ribeiro, N. Garcia-Porta, J. M. González-Meijome, *Contact Lens Anterior Eye* **2014**, *37*, 240.

[61] J. C. Bavinger, K. DeLoss, S. I. Mian, *Curr. Opin. Ophthalmol.* **2015**, *26*.

[62] S. La Porta Weber, R. Becco de Souza, J. Á. P. Gomes, A. L. Hofling-Lima, *Am. J. Ophthalmol.* **2016**, *163*, 167.

Supplementary Figures and Tables

Functional Contact Lenses	Functionalities					
	Sensing		Antimicrobial		Enhanced Optical Properties	
	Pathology (Analyte(s)/Parameter(s))	Mode	Bacteria Tested	Mode	Optical Property	Mode
Research						
**Ruan *et al.* (2017)**, doi: 10.3390/polym9040125	Diabetes (Glucose)	Nanophotonics	X	X	X	X
**Elsherif *et al.* (2018)**, doi: 10.1021/acsnano.8b00829	Diabetes (Glucose)	Nanophotonics	X	X	X	X
**Kim *et al.* (2020)**, doi: 10.1038/s41598-020-65103-z	Diabetes (Glucose)	Nanophotonics	X	X	X	X
**Maeng *et al.* (2020)**, doi: 10.1039/c9lc01268k	Glaucoma (IOP)	Nanophotonics	X	X	X	X
**Karepov & Ellenbogen (2020)**, doi: 10.1364/OL.401507	Color Blindness ( )	Nanophotonics	X	X	X	X
**Wang *et al.* (2020)**, doi: 10.1039/C9TB02389E	Xerophthalmia and Glaucoma (Water content and IOP)	Nanophotonics	X	X	X	X
**Badawy *et al.* (2018)**, doi: 10.1002/adhm.201800152	Color Blindness ( )	Functionalization	X	X	X	X
**Riaz *et al.* (2019)**, doi: 10.1021/acsomega.9b02638	(pH)	Functionalization	X	X	X	X
**Moreddu *et al.* (2019)**, doi: 10.1039/C9RA00601J	(Temperature)	Functionalization	X	X	X	X
**Yetisen *et al.* (2020)**, doi: 10.1002/adma.201906762	Dry Eye (Electrolytes and pH)	Functionalization	X	X	X	X
**Choi *et al.* (2020)**, doi: 10.1021/acsnano.9b10145	Dry Eye (Reactive Oxygen Species)	Functionalization	X	X	X	X
**Thomas *et al.* (2012)**, doi: 10.1016/j.snb.2011.12.049	(Lactate)	Electrical/Electrochemical	X	X	X	X
**Lee *et al.* (2017)**, doi: 10.1021/acsnano.7b00370	Dry Eye (EM Shielding and Anti-Dehydration)	Electrical/Electrochemical	X	X	X	X
**Kim *et al.* (2017)**, doi: 10.1038/ncomms14997	Diabetes and Glaucoma (Glucose and IOP)	Electrical/Electrochemical	X	X	X	X
**Chiou *et al.* (2017)**, doi: 10.3390/s17010108	Glaucoma and Dry Eye (IOP and Hydration Level)	Electrical/Electrochemical	X	X	X	X
**Park *et al.* (2018)**, doi: 10.1126/sciadv.aap9841	Diabetes (Glucose)	Electrical/Electrochemical	X	X	X	X
**Yin *et al.* (2018)**, doi: 10.1126/sciadv.aap9841	(Electroretinography)	Electrical/Electrochemical	X	X	X	X
**Kusama *et al.* (2020)**, doi: 10.1002/admt.201900889	Dry Eye (Anti-Dehydration)	Electrical/Electrochemical	X	X	X	X
**Ku *et al.* (2020)**, doi: 10.1126/sciadv.abb2891	Stress (cortisol)	Electrical/Electrochemical	X	X	X	X
**Jang *et al.* (2021)**, doi: 10.1126/sciadv.abf7194	Ocular surface inflammation (MMP-9)	Electrical/Electrochemical	X	X	X	X
**Keum *et al.* (2020)**, doi: 10.1126/sciadv.aba3252	Diabetes (Glucose)	Electrical/Electrochemical	X	X	X	X
**Bazzaz *et al.* (2014)**, doi: 10.1016/j.clae.2013.09.008	X	X	*P. aeruginosa, S. aureus*	Ag Nanoparticles	X	X
**Tuby *et al.* (2016)**, doi: 10.1002/cnma.201600066	X	X	*P. aeruginosa, S. epidermidis*	Zn-Cuo Nanoparticles	X	X
**Liu *et al.* (2018)**, doi: 10.1021/acsbiomaterials.7b00977	X	X	*P. aeruginosa, A. fumigatus*	Ag Nanoparticles	X	X
**Lai *et al.* (2018)**, doi: 10.1039/C7RA12753G	X	X	X	X	UV/blue-light reflection	Nanostructures
Industry						
**Alphabet Inc./Alcon** (2014 - Present)	Diabetes and Presbyopia (Glucose and Vision Accommodation)	Electrochemical	X	X	X	X
**Sensimed AG** (2010 - Present)	Glaucoma (IOP)	Electrochemical	X	X	X	X

Table S1. Summary of the literature survey on different functional contact lenses developed both in research and industry. Functionalities include sensing/actuation, antimicrobial and

enhanced optical properties. Red X indicates absence of functionality in the device. Table is color coded based on sensing modality.

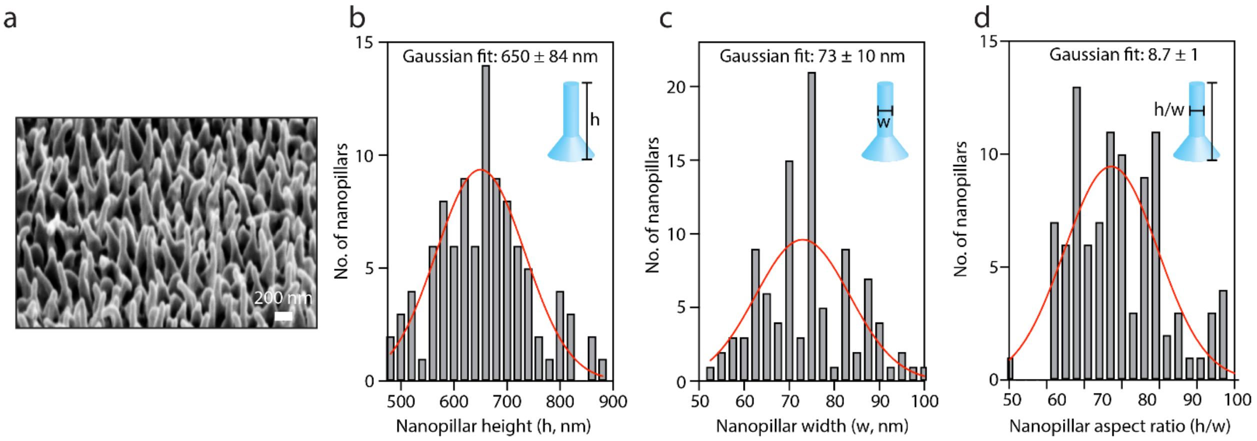

Figure S1. Characterization of *G. oto* nanostructures. (a) SEM image of *G. oto* nanostructures. Nanostructure (a) height, (c) width, and (d) aspect-ratio modeled using a Gaussian distribution.

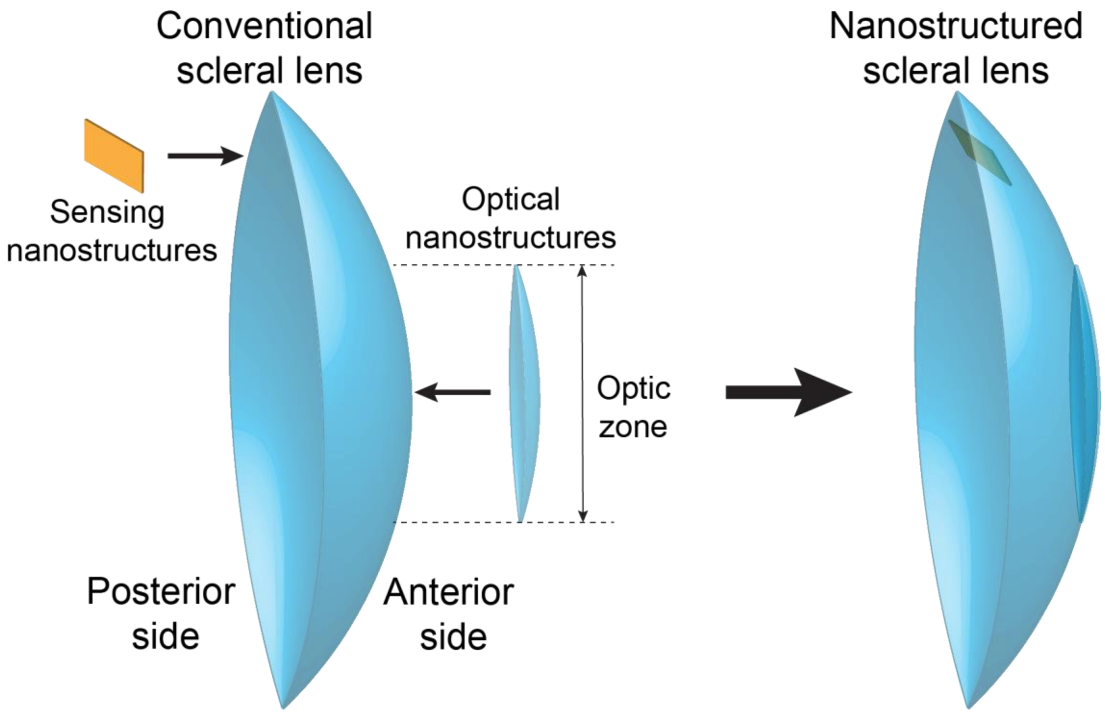

Figure S2. Integration of the optical/bactericidal and sensing nanostructured parylene films onto a conventional scleral lens. A section of the clear optical nanostructured parylene film is bonded to the anterior side of the scleral lens in the center of the optic zone using UV-curable epoxy. Next, a section of the Au-coated sensing nanostructured parylene film is bonded in the same fashion but beyond the periphery of the optic zone on the posterior side of the same lens.

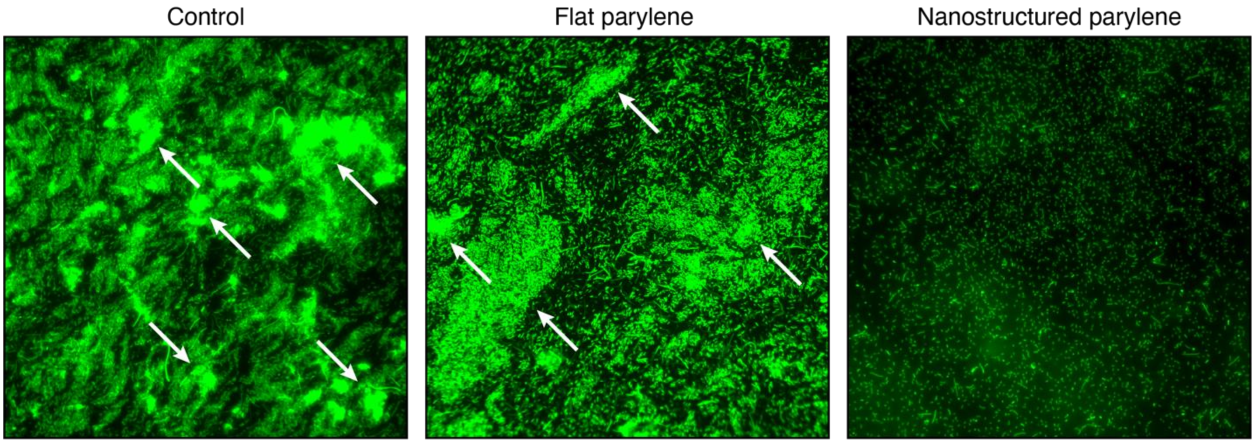

Figure S3. Fluorescent micrographs of SYTO 9 showing early signs of biofilm formation as bacterial microcolonies (indicated by white arrows) on the control and flat parylene surfaces after 4 hr.

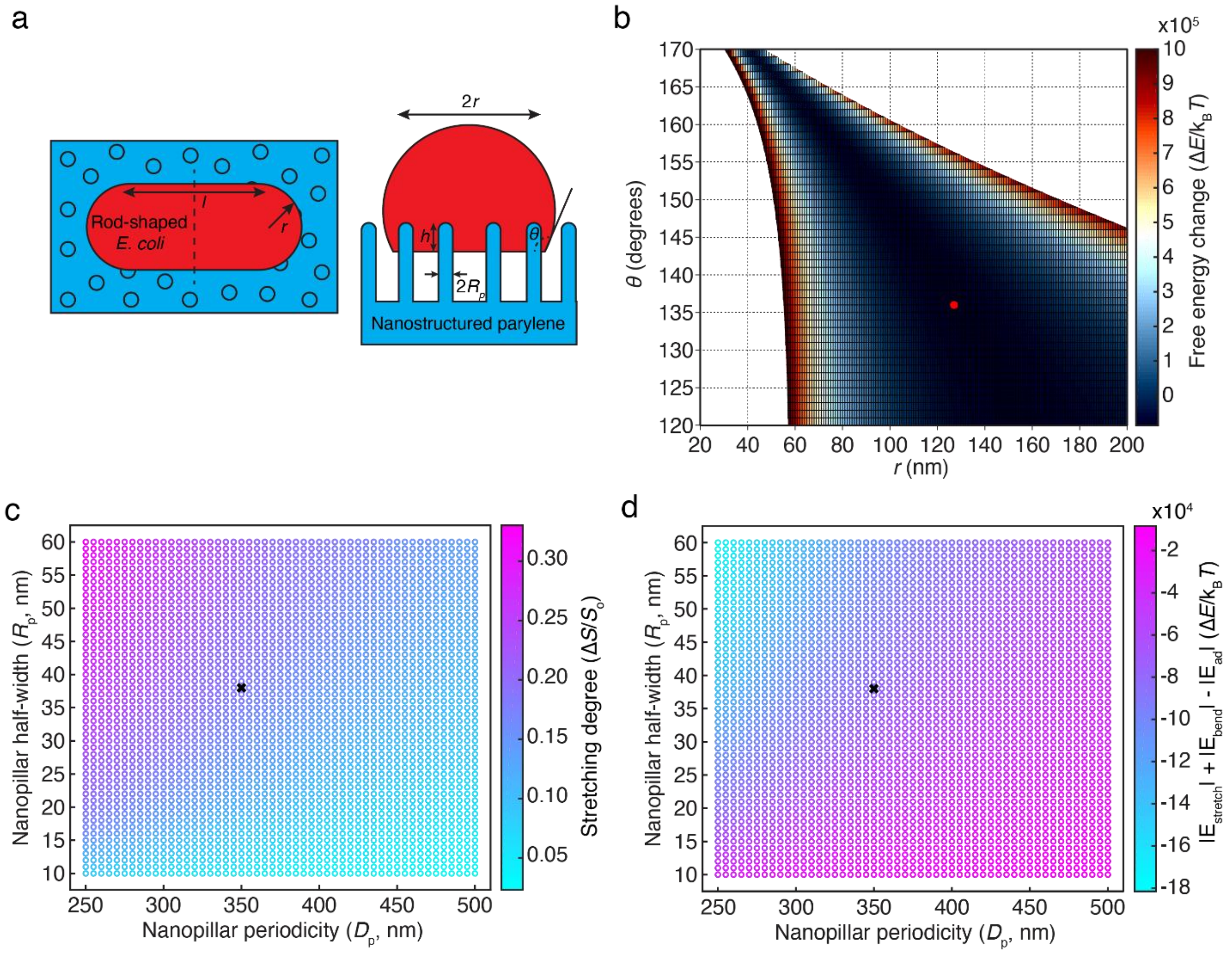

Figure S4. Thermodynamic modeling of *E. coli* adhesion on the nanostructured parylene surface. (a) Schematic of the model. (b) Minimization of the free energy change ΔE as a function of (r,θ) for an *E. coli* bacterium on the parylene nanostructures estimating their average half-width (R_p = 37.5 nm) and short-range periodicity ($\sim D_p$ = 350 nm). The red dot corresponds to the global minima of ΔE. (c) Stretching degree ($\frac{\Delta S}{S_o}$) computed for various

combinations of nanostructure half-width (R_p) and periodicity (D_p). The black cross corresponds to the $\frac{\Delta S}{S_0}$ obtained for the parylene nanostructures given their average R_p and D_p. (d) $|E_{stretch}| + |E_{bend}| - |E_{ad}|$ computed for various combinations of nanopillar half-width (R_p) and periodicity (D_p). The black cross corresponds to the $|E_{stretch}| + |E_{bend}| - |E_{ad}|$ obtained for the parylene nanostructures given their average R_p and D_p.

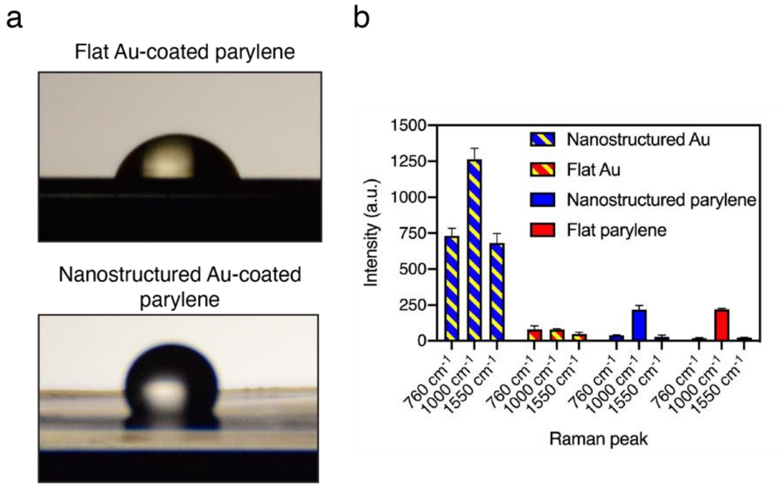

Figure S5. DCDRS enhancement due to increased hydrophobicity. (a) A water droplet on a flat Au-coated parylene (CA = 67°) and Au-coated nanostructured parylene (CA = 122°). (b) Increased hydrophicity leads to ~10x DCDRS enhancement when measuring 3 mg/mL lysozyme in PBS.

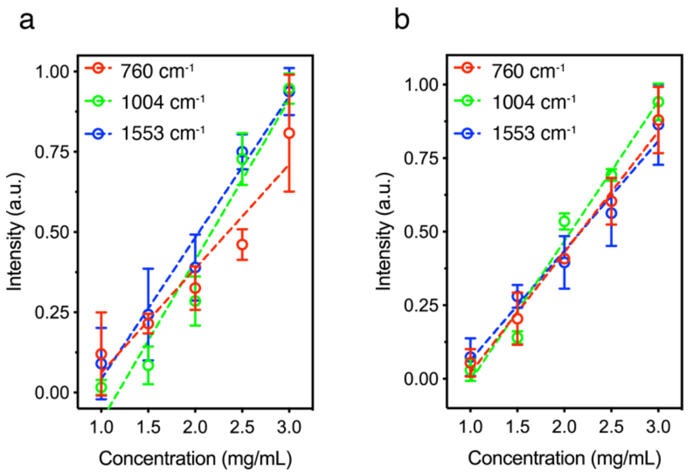

Figure S6. Normalized DCDRS intensity for (a) lactoferrin and (b) lysozyme in PBS between 1 – 3 mg/mL concentrations. The 760, 1004 and 1553 cm^{-1} peaks are tracked.

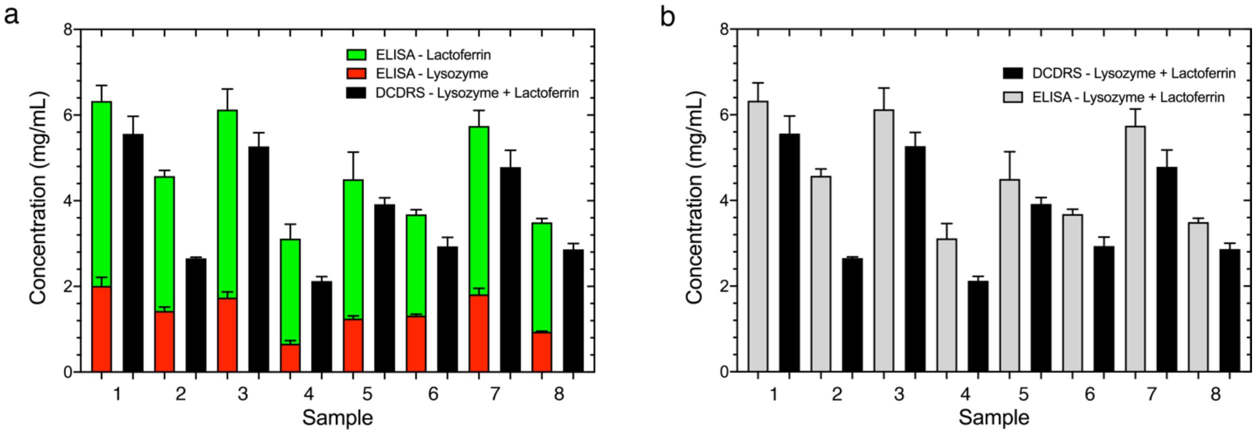

Figure S7. ELISA and scleral lens sensor on whole tear samples. (a) Concentrations of lactoferrin and lysozyme measured separately using ELISAs. (b) Aggregated concentration for ELISA compared with that measured using the scleral lens sensor prior to sensor calibration. The results from the scleral lens sensor is consistently lower.
